# The improvement of the in vitro plant regeneration in barley with the epigenetic modifier of histone acetylation, trichostatin A

**DOI:** 10.1007/s13353-023-00800-9

**Published:** 2023-11-14

**Authors:** Katarzyna Nowak, Barbara Wójcikowska, Monika Gajecka, Anna Elżbieciak, Joanna Morończyk, Anna M. Wójcik, Przemysław Żemła, Sylvie Citerne, Agnieszka Kiwior-Wesołowska, Justyna Zbieszczyk, Małgorzata D. Gaj

**Affiliations:** 1 Faculty of Natural Sciences, Institute of Biology, Biotechnology and Environmental Protection, University of Silesia, 40-007 Katowice, Poland; 2 Toxicology Research Group, Łukasiewicz Research Network, Institute of Industrial Organic Chemistry Branch Pszczyna, Doświadczalna 27, 43-200 Pszczyna, Poland; 3 Institut Jean-Pierre Bourgin (IJPB), INRAE, AgroParisTech, Université Paris-Saclay, 78000 Versailles, France

**Keywords:** Barley, Plant regeneration, Trichostatin A, Hormones level, Transcription factors

## Abstract

Genotype-limited plant regeneration is one of the main obstacles to the broader use of genetic transformation in barley breeding. Thus, developing new approaches that might improve responses of in vitro recalcitrant genotypes remains at the center of barley biotechnology. Here, we analyzed different barley genotypes, including “Golden Promise,” a genotype commonly used in the genetic transformation, and four malting barley cultivars of poor regenerative potential. The expression of hormone-related transcription factor (TF) genes with documented roles in plant regeneration was analyzed in genotypes with various plant-regenerating capacities. The results indicated differential expression of auxin-related *TF* genes between the barley genotypes in both the explants and the derived cultures. In support of the role of auxin in barley regeneration, distinct differences in the accumulation of free and oxidized auxin were observed in explants and explant-derived callus cultures of barley genotypes. Following the assumption that modifying gene expression might improve plant regeneration in barley, we treated the barley explants with trichostatin A (TSA), which affects histone acetylation. The effects of TSA were genotype-dependent as TSA treatment improved plant regeneration in two barley cultivars. TSA-induced changes in plant regeneration were associated with the increased expression of auxin biosynthesis-involved *TFs*. The study demonstrated that explant treatment with chromatin modifiers such as TSA might provide a new and effective epigenetic approach to improving plant regeneration in recalcitrant barley genotypes.

**Supplementary Information:**

The online version contains supplementary material available at 10.1007/s13353-023-00800-9.

## Introduction

Barley (*Hordeum vulgare* L.) is the fourth most important cereal in the world after corn (*Zea mays*), rice (*Oryza sativa*), and wheat (*Triticum aestivum*). In 2021, barley world production reached 145 million tons, with a global area of spring barley cultivation of almost 50 million hectares (FAO, https://www.fao.org/faostat/en/#home). The high starch and protein content in barley grains predisposes this cereal to be used in different industries, including brewing and food production for animals and humans (Baik and Ullrich 2008) and recombinant protein production (Yemets et al. 2011).

A long history of cultivation and adaptation to a wide range of agro-climatic conditions resulted in a huge genetic diversity of barley that was estimated by SNPs for 60–90% based on analysis of 96 spring barley cultivars and 260 Ethiopian barley accessions (Bykova et al. 2017; Teklemariam et al. 2022). Barley cultivars and breeding lines show great plant and grain morphology diversity and tolerance to environmental stresses, including resistance to temperature and drought (Dawson et al. 2015; Jabbari et al. 2018). In addition to a high position in plant breeding, and due to a diploid chromosome number, self-pollination, and public access to the genome sequence, barley has also been recommended as a model plant for research on the Triticeae tribe, such as polyploid wheat or rye (*Secale cereale*) (Saisho and Takeda 2011).

In recent years, genetic transformation has become a central tool in the research and breeding of crops, including cereals (Anjanappa and Gruissem 2021). A prerequisite for implementing genetic transformation protocol is the efficient regeneration of plants from transgenic tissues. The strong genotypic effect on in vitro plant regeneration substantially limits the routine use of modern biotechnology tools in some crops, including cereals and among them barley (Lü et al. 2015; Hayta et al. 2019; Debernardi et al. 2020).

In barley, the routine production of transgenic lines in the culture of somatic tissue is limited to a few genotypes, and “Golden Promise” (GP) is the most commonly used in agrotransformation (Matres et al. 2021). However, inferior agronomic characteristics compared to modern elite barley cultivars, including a low yield and sensitivity to diseases, particularly powdery mildew, substantially limit use of GP in breeding programs (Douchkov et al. 2014). Genetic transformation for a few other genotypes, including “Scarlett”, was also reported but with limited success (Nadolska-Orczyk et al. 2000; Zalewski et al. 2012). Unfortunately, most barley cultivars remain recalcitrant to genetic transformation due to the low plant regeneration capacity (Harwood 2012; Orman-Ligeza et al. 2020).

Besides genotype, the effectiveness of plant regeneration in vitro is affected by various exo- and endogenous factors (Bidabadi and Mohan Jain 2020). In barley, the optimization of in vitro culture protocols involved chemical and physical culture conditions, in particular, the composition of media, including the macro- and micronutrients, the plant-growth regulators (PGRs), and the explant type (Dahleen and Bregitzer 2002; Chauhan and Kothari 2004; Ganeshan et al. 2006). In barley protocols, the callus induced from the explants on CIM (callus induction medium) is transferred onto PRM (plant regeneration medium) to regenerate plants. The CIM and PRM media substantially differ in PGR composition, and depending on the protocol, various PGRs have been recommended, including Dicamba, 6-benzylaminopurine (BAP), or 2,4-dichlorophenoxyacetic acid, to regenerate barley plants efficiently (Chang et al. 2003; Serhantova et al. 2004; Hensel et al. 2009). In line with media optimization, different explants have been evaluated, mature and immature zygotic embryos (IZEs) at different developmental stages and embryo fragments have been recommended for effective plant regeneration (Chang et al. 2003; Senarath 2007; Hensel et al. 2009).

Plant regeneration from the explant cells is a multi-staged developmental process controlled by a complex network of genetic and epigenetic factors (Bidabadi and Mohan Jain 2020). Studies on a model plant of Arabidopsis and other species revealed transcription factors (TFs), miRNAs, DNA methylation, and histone modifications to play a central role in plant regeneration in vitro (Shin et al. 2020; Wójcikowska et al. 2020; Salaün et al. 2021). In particular, TFs of regulatory functions in gene expression have been widely documented to control in vitro responses of plants (Salaün et al. 2021). Although hundreds of *TFs* of differential expression in plant regeneration were reported (Gliwicka et al. 2013; Wickramasuriya and Dunwell 2015; Lardon et al. 2020; Suo et al. 2021; Xu et al. 2022), only a few were functionally analyzed in terms of their role in the plant regeneration mechanism. The studies on Arabidopsis revealed the functions of several *TFs*, including *BABY BOOM*-*BBM* (Boutilier et al. 2002), *LEAFY COTYLEDON1* and *2*-*LEC1* and *LEC2* (Lotan et al. 1998; Gaj et al. 2005), *WUSCHEL*-*WUS* (Zuo et al. 2002), *AGAMOUS-LIKE15*-*AGL15* (Harding et al. 2003), *MYB118* (Wang et al. 2009), and *EMBRYOMAKER*-*EMK* (Tsuwamoto et al. 2010), in developmental cell reprogramming. Some of these TFs were also reported to be involved in the plant regeneration of other species, including barley (Heidmann et al. 2011; Belide et al. 2013; Guo et al. 2013; Brand et al. 2019; Suo et al. 2021). However, further analysis is necessary to identify the main regulators of plant regeneration in cereals including barley.

The advances in deciphering genetic networks controlling plant regeneration in vitro resulted in the development of a new “genetic strategy” to overcome limitations in plant regeneration of recalcitrant species (Lee and Wang 2023). Following this approach, transgenic lines that overexpressed *TF* genes, including *BBM*, *LEC2*, and efficiently regenerated plants, have been produced in *Populus tomentosa*, *Capsicum annuum*, and *Brassica napus* (Deng et al. 2009; Heidmann et al. 2011; Belide et al. 2013).

Here, we propose an “epigenetic approach” for improving plant regeneration in recalcitrant barley genotypes. We aimed to modify the epigenome of barley explant cells by treatment with an antifungal antibiotic, trichostatin A (TSA), of inhibitory effect on histone deacetylases (HDACs) (Tsuji et al. 1976; Furumai et al. 2001). Since HDAC enzymes remove the acetyl group from the ε-amino group of the lysine side chains (Seto and Yoshida 2014), TSA promotes the hyperacetylation of histones that might result in an open chromatin state and the transcription of genes, including those controlling plant regeneration (Görisch et al. 2005). In line with this assumption, TSA promotes the development of embryogenic tissue in seedlings (Tanaka et al. 2008), and in vitro cultured explants of Arabidopsis (Wójcikowska et al. 2018) and conifers (*Picea abies* and *Pinus sylvestris*) (Uddenberg et al. 2011; Abrahamsson et al. 2017). In addition to the vegetative explants, the beneficial effects of TSA on the microspore cultures of *T. aestivum* (Jiang et al. 2017) and *B. napus* (Li et al. 2014) have been documented. Interestingly, TSA treatment might recompense the need for auxin treatment, and TSA-treated Arabidopsis explants (IZEs) regenerated plants via somatic embryogenesis (SE) on a medium free of auxin, a key inducer of embryogenic transition (Wójcikowska et al. 2018). Thus, here we investigated the effect of TSA on plant regeneration of barley cultivars recalcitrant for the in vitro culture.

## Materials and methods

### Plant material

Five spring barley cultivars with divergent pedigrees were used: GP (UK) and four malting cultivars: “Morex” (USA), “Scarlett” (Germany), “Krona” (Germany), and “Dema” (Poland), except for the six-row cv. “Morex”, the rest of the cultivars are two-row. The barley cultivars have diverse genetic backgrounds. The malting cultivars resulted from the following crosses: “Dema”–“Aramir” × “Georgia”; “Krona”–(“Nebi” × “Trumpf”) × (“Union” × “Gimpel”); “Morex”–“Cree” × “Bonanza”; “Scarlett” × (“Amazone” × “Breun St. 2730”) × “Kym.” (Russell et al. 1997; https://grinczech.vurv.cz/gringlobal/search.aspx; https://beerandbrewing.com/dictionary/HzSYZgMg59/). The GP was a gamma-ray-induced mutant of “Maythorpe” (Russell et al. 1997).

### Plant growth and in vitro culture conditions

The donor plants for the in vitro culture were grown at 18/16 °C for 3 weeks in a growth room at a 200 μM s^−1^ m^−2^ light intensity. Then the plants were transferred to a growth chamber with 17/14 °C day/night temperature conditions, 480–500 μM s^−1^ m^−2^ photon flux density of illumination, and 16/8 h photoperiod. The plant materials grown in sterile in vitro conditions were kept at 24 °C under a 16/8 h photoperiod of 40 μM m^−2^ s^−1^ white, fluorescent light.

### Explants for in vitro culture

Seeds with IZEs of approximately 1.5–2 mm diameter were collected from the pot-growing plants, washed with 70% ethanol for 3 min with shaking, and then rinsed once with sterile distilled water. Then seeds were surface-sterilized in a solution of sodium hypochlorite (2.4%) containing a few drops of Tween20 for 15 min with shaking. Afterward, seeds were rinsed five times (1 min) in sterile, distilled water. The IZEs were isolated from the seeds, and the embryo axes were removed to isolate scutella, as described by Marthe et al. (2015). The scutella were used as explants for TSA treatment and in vitro culture.

### Callus induction and regeneration

Ten isolated scutella were cultured in a Petri dish with an agar CIM supplemented with 2.5 mg/L Dicamba (Sigma-Aldrich, St. Louis, MO, USA) (Supplementary Table 1) in dark conditions. After 2 weeks, scutella-derived calli were transferred to a fresh CIM medium for the next 2 weeks. Then, the explants were transferred to plant regeneration (K4NB) medium-PRM with a 0.225 mg/L BAP (Sigma-Aldrich, St. Louis, MO, USA) (Supplementary Table 1) and cultured in the light (growth conditions described in a section on “Plant growth and in vitro culture conditions”). The medium was refreshed every 2 weeks, and after 4 weeks on the PRM medium, the explant capacity for plant regeneration was analyzed.

### Explants treatment with TSA

To analyze the effect of TSA, an inhibitor of HDACs, on the plant regeneration capacity of explants, the CIM medium was supplemented with trichostatin TSA (Sigma Aldrich; St. Louis, MO, USA #T1952) at concentrations of 1.0, 2.5, 5.0, and 7.5 μM. The explants were cultured on the TSA-supplemented CIM medium for 1, 2, and 4 weeks.

### Evaluation of regeneration capacity

The capacity for plant regeneration of 8-week-old cultures was evaluated. Plant regeneration efficiency (the percentage of explants that formed shoots) and plant regeneration productivity (the average number of shoots produced per regenerating explant) were scored. Each culture combination was evaluated in at least three replicates, and 30 explants (ten explants/Petri dish) were analyzed per one replicate.

### Histological examination

The explants of “Golden Promise” cultured on PRM medium (35 and 42 days) were subjected to histological examination. The plant tissue was fixed in a mixture of 3% (w/v) paraformaldehyde (Sigma-Aldrich, St. Louis, MO, USA) and 1.25% (v/v) glutaraldehyde (Sigma-Aldrich, St. Louis, MO, USA) in PBS (phosphate-buffered saline, pH 7.2). Samples were de-aerated in fixative for 2 h, and incubated in fixative overnight at 4 °C. After rinsing with PBS (3 × 20 min), the plant material was dehydrated in an ethanol series (10, 30, 50, 70, 90, and 100%) and embedded in Steedman’s wax. The blocks with plant tissue were cut (for 8 μm thick) using a Zeiss HYRAX M40 rotary microtome (Jena, Germany) and collected on microscopic slides covered with poly-L-lysine (Menzel Gläser, Germany). The sections were dewaxed, rehydrated in a series of ethanol solutions (3 × 100%, 1 × 90%, 1 × 50% (v/v) solution, 1× distilled water; each wash for 10 min), and prepared for the histochemical analysis. We used two different staining methods, PAS (periodic-acid-Schiff) reaction and sequenced standing with methyl and toluidine blue. The sections were oxidized in a 0.5% aqueous periodic acid (Sigma-Aldrich, St. Louis, MO, USA) solution for 1 h (room temperature), washed in running water (10 min), and then rinsed with distilled water. The slides were placed in Schiff’s reagent (Sigma-Aldrich, St. Louis, MO, USA) (15 min) in darkness, rinsed with distilled water, and transferred to a 0.5% sodium sulfite solution (2 min). After washing with running tap water (10 min), sections were placed in a 0.5% Toluidine Blue 0 aqueous solution (5 s) to visualize the meristematic cells. The sections were dehydrated in an ethanol series (20% and 40% ethanol for 2 min, 60%, 80%, and 100% for 3 min). For another method, the sections were stained with 0.1% (w/v) methyl blue (Sigma-Aldrich, St. Louis, MO, USA) in a 0.15 M K_2_HPO_4_ solution (10 min), rinsed three times with distilled water. They were then incubated in 0.01% Toluidine Blue 0 (in PBS) for 10 min, rinsed with distilled water, and mounted in Fluoromount (Sigma-Aldrich, St. Louis, MO, USA). All observations and photography were done using a Keynote VHX-6000 (Keynote, Mechelen, Belgium) with corresponding software.

### Phytohormones concentration measurements

The concentration of phytohormones, including free IAA — free indole-3-acetic acid, IAAox — 2-oxindole-3-acetic acid, JA — jasmonic acid, ABA — abscisic acid, and SA — salicylic acid, was evaluated in the freshly isolated (0 day), and 14 days, 28 days (CIM medium), and 35 days (PRM medium) cultured explants of GP, “Scarlett,” and “Dema.” For analysis, fresh tissue (at least 100 mg) was immediately frozen in liquid nitrogen and stored at −80 °C. Then, they were freeze-dried and ground. The phytohormone concentrations were determined, as described by Li-Marchetti et al. (2015). For each sample, 7 mg of dry powder was extracted with acetone/water/acetic acid (80/19/1). IAA, ABA, SA, and JA stable labeled isotopes used as internal standards were prepared, as described by Le Roux et al. (2014). The experiment was carried out in triplicate.

### Analysis of gene expression

Gene expression was analyzed in control and TSA-treated (7.5 μM TSA for 4 weeks on CIM medium) cultures of GP, “Scarlett,” and “Dema.” Total RNA was isolated from cultures induced on the CIM (for 0, 7, 14, 21, and 28 days) and the PRM (for 35 days) medium. To isolate the RNA from the 0 day culture, scutella of IZEs were collected in RNAlater (Life Technologies, Carlsbad, CA, USA) and then treated the same way as the explants that had been cultured on CIM and PRM medium. Total RNA was isolated using a miRVana miRNA Isolation Kit (Thermo Fisher Scientific, Waltham, MA, USA). Depending on the age of the culture, 100 (0 day) to 5 (35 days) explant-derived cultures were used for RNA isolation in one biological replicate. RNA concentrations were measured using a Nano-Drop ND-1000 (NanoDrop Technologies, Wilmington, DE, USA). One microgram of total RNA per sample was treated with RQ1 RNase-Free DNase (Promega Medison, WI, USA) and reverse transcribed in a 20 μl reaction volume using a RevertAid First Strand cDNA Synthesis Kit (Thermo Fisher Scientific, Waltham, MA, USA) with oligo-dT primers, according to the manufacturer’s instructions. The obtained cDNA was diluted fourfold with water and used at a volume of 2.0 μl in a qPCR reaction. Analyses were performed in a 10 μl volume using a LightCycler® 480 SYBR Green I Master (Roche, Basel, Switzerland) in two technical repeats. The primers used in the analyses were designed with Primer3Plus (Supplementary Table 2). The reference gene used in this study was *EF1* (elongation factor 1-α; Rapacz et al. 2012). Analyses were performed using a LightCycler 480 (Roche, Basel, Switzerland) under the following reaction conditions: initial denaturation of 5 min at 95 °C, followed by 10 s at 95 °C, 20 s at a temperature specific for the primers, 10 s at 72 °C, repeated in 40 cycles. Denaturation for the melt curve analysis was conducted for 5 s at 95 °C, followed by 1 min at 65 °C and heating to 98 °C (0.1 °C/s for the fluorescence measurement). The Ct values and the value of the qPCR efficiency were obtained from LinRegPCR (version 11, Academic Medical Centre, Amsterdam, The Netherlands). The plant tissues for the real-time qPCR analysis were produced in three biological repetitions. The relative expression level was calculated using 2^−∆∆CT^, where ∆∆C_T_ represents ∆C_T_^reference condition^ − ∆C_T_^compared condition^.

### Statistical analysis

The two-way ANOVA (*p* < 0.05) followed by Tukey’s honestly significant difference test (Tukey HSD-test) (*p* < 0.05) was used to calculate any significant differences between the experimental combinations. The graphs show the average values with the standard deviation (SD).

## Results

### Plant regeneration efficiency in barley is highly genotype-dependent

#### The malting cultivars (“Morex,” “Scarlett,” “Krona,” and “Dema”) have significantly lower plant regeneration efficiency than GP

**Fig. 1.**
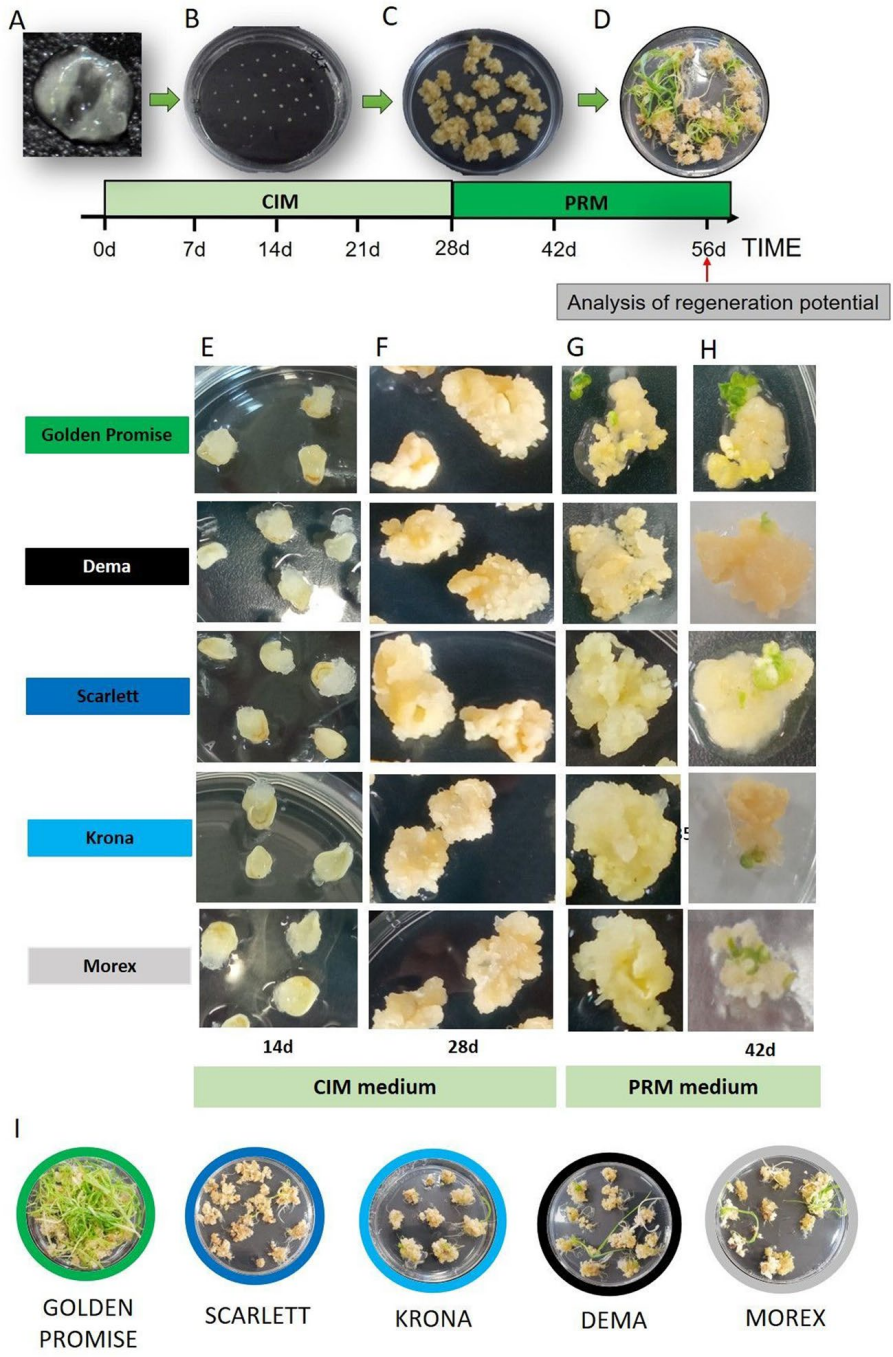
Plant regeneration in barley following the protocol of Hensel et al. (2009) in which scutella of immature zygotic embryos were used as explants. The freshly isolated 0 day explants (**A**) and explants cultured for 14 days (**B**), 28 days (**C**) on the CIM medium, and 56 days (**D**) on the PRM medium. The timeline represents the culture time points selected for gene expression and hormone concentration analysis. The induction of callus tissue on CIM medium (**E**, **F**) and plant regeneration on PRM medium (**G**, **H**) in the culture of different barley cultivars: “Golden Promise”, “Dema”, “Scarlett”, “Krona”, and “Morex”. The cultures at 14 (**E**), 28 (**F**), 35 (**G**), 42nd (**H**), and analysis day — 56 days (**I**) were shown

We evaluated the plant regeneration potential of different barley cultivars, including GP, “Morex”, “Scarlett”, “Krona”, and “Dema”, following the culture protocol of Hansel and colleagues (2009). We used the GP cultivar of high plant regenerative potential as a reference genotype. The explants, scutella of IZEs, were induced for 4 weeks on the auxin-rich CIM medium to produce calli. Then, the calli were cultured for the next 4 weeks on the cytokinin-supplemented PRM medium (Fig. 1A–D). The cultures induced on CIM and PRM medium were inspected and sampled along the culture time, and morphogenic processes in callus of different genotypes were analyzed at macroscopic and histological levels. Callus tissue appeared macroscopically on explants in all barley genotypes on the 7th day of CIM culture. White-yellowish callus tissue was produced along the edges of the scutella in place of the embryonic axis that was removed from the explant (Fig. 1E). During the next 3 weeks on the CIM, the callus production increased, and the tissue tended to separate into numerous, small tissue clumps (Fig. 1F). In conclusion, the timing of induction, the dynamic of growth, and the morphology of the callus seemed to be similar in different barley genotypes.

In contrast to CIM_,_ which similarly affected different genotypes, differences in timing and efficiency of morphogenic responses between GP and other genotypes were indicated in culture on the PRM. In GP, a 7-day callus induction on the PRM (35 days of culture) resulted in the first patches of greenish tissue, suggesting the induction of the shoot regeneration process (Fig. 1G). Consequently, in the following week (42 days of culture), numerous shoots were produced in the GP culture (Fig. 1H). In contrast to GP, other genotypes showed delayed timing of shoot regeneration. Calluses of “Morex”, “Scarlett”, “Krona”, and “Dema” could produce the first shoots at least 1 week later than GP (Fig. 1H). The 8-week-old cultures were analyzed regarding plant regeneration potential (Fig. 1I).

**Fig. 2.**
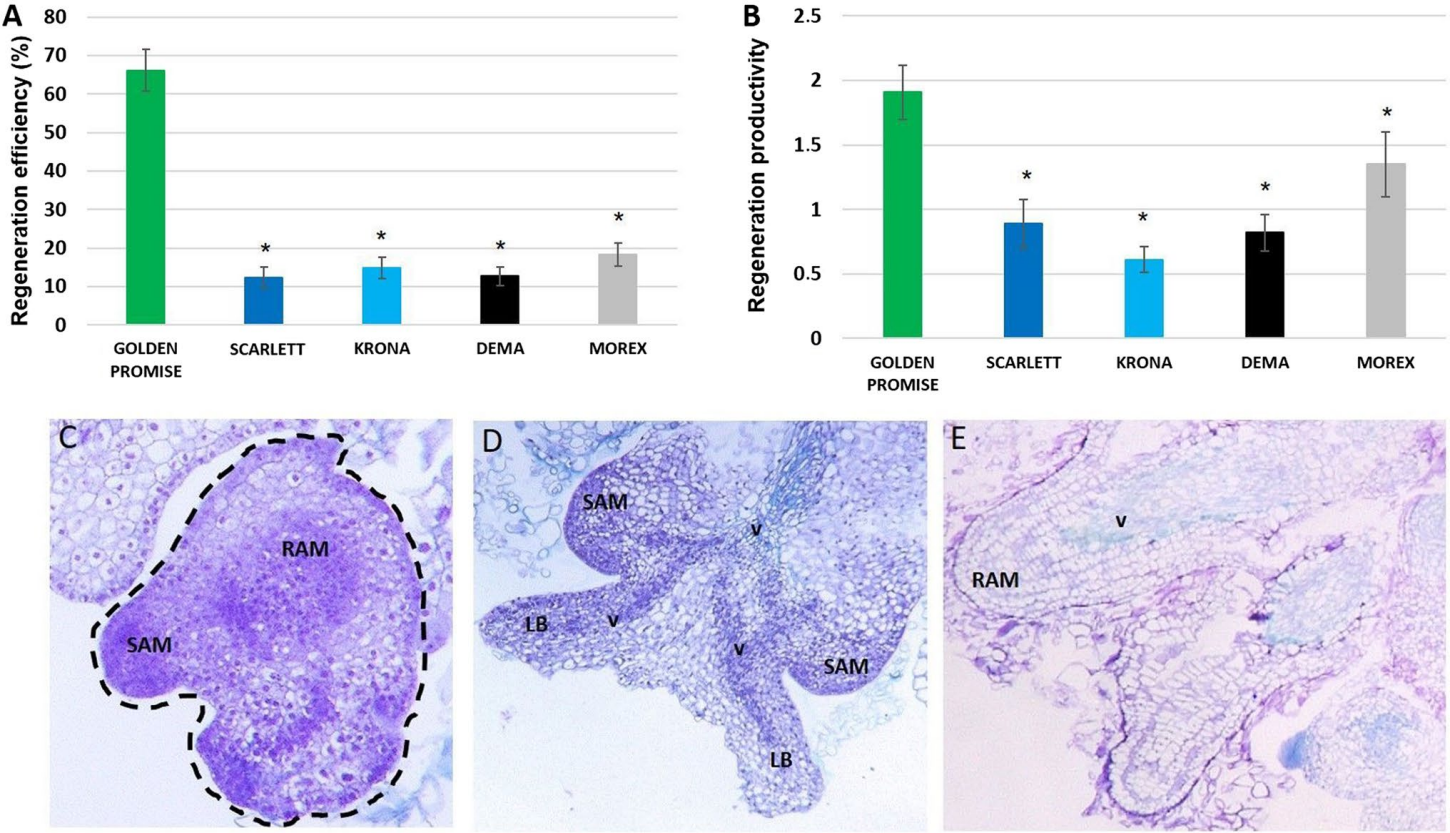
Plant regeneration potential of different barley cultivars: “Golden Promise”, “Scarlett”, “Krona”, “Dema”, and “Morex”. Plant regeneration efficiency (**A**) and productivity (**B**) of culture. * — values significantly different from “Golden Promise” (*p* < 0.05; *n* = 3; means ± SD are given). Histological analysis of the “Golden Promise” culture: somatic embryos at 35 days of culture (**C**) and regeneration of shoots (**D**) and roots (**E**) at 42 days of culture. Different types of structures were marked: SAM (shoot apical meristem), LB (leaf bud), v (vascular bundle), and RAM (root apical meristem). The sections were stained with methyl and toluidine blue (**C**, **D**) and PAS (**E**)

The effectiveness of callus production on CIM and PRM in different genotypes was quantified. All genotypes were highly effective in callus production, and 100% of explants developed callus. The calli of different genotypes induced on the PRM showed a significantly different capacity for shoot production that was evaluated by the frequency of explants producing at least one shoot (regeneration efficiency, Fig. 2A) and the average number of shoots produced per regenerated explant (regeneration productivity, Fig. 2B). The results indicated the highest plant regeneration potential of the GP culture, and 65% of the GP explants regenerated shoots with over 1.9 productivity on average. Explants of other genotypes regenerated shoots with over three to five times lower efficiency ranging from 12% (“Scarlett”) to 18% (“Morex”). These genotypes also showed lower than GP plant regeneration productivity, and their explants produced 0.6–1.3 shoots per explant.

To identify the morphogenic pathways involved in the regeneration of barley plants on the PRM medium, the GP callus were histologically examined on the 35th and 42nd day of culture (Fig. 2C–E). Callus was stained with PAS to identify callose in cell walls which marks newly formed cells, including those with embryogenic potential (Dubois et al. 1991). In addition, aniline and toluidine blue staining were used to identify meristematic/embryogenic callus cells with the potential for plant regeneration (O’Brien et al. 1964).

The histological examination provided evidence that the regeneration of barley plants in the GP explants might proceed through two alternative pathways, SE and shoot organogenesis (ORG) (Fig. 2C–E). Accordingly, the bipolar embryo-like structures with shoot apical meristem and a root pole were found in the 35-day-old culture (Fig. 2C). These structures were not connected to the explant through vascular tissue, and this supported an assumption about their embryo-like feature. Besides bi-, unipolar structures connected with the explants and resembling shoot meristems with leaf buds and root meristems were found in 42 days culture (Fig. 2D,E). These structures implied a shoot and root ORG process to be induced in barley calli.

In summary, the barley genotypes showed a high capacity of explants for callus production. However, the capacity of calli for shoot regeneration was highly genotype-dependent. Most of the cultivars, including “Morex”, “Scarlett”, “Krona”, and “Dema”, indicated much lower effectiveness for shoot regeneration than the GP model cultivar. Thus, genetic/epigenetic differences in controlling plant regeneration in barley are assumed.

#### The genes controlling plant regeneration show distinctly different expressions in GP vs. other genotypes

We inquired whether different plant regeneration capacities of the barley genotypes may be related to differences in the expression of genes controlling plant regeneration. We profiled the expression of analyzed genes encoding TFs such as *LEC1*, *FUS3*, *BBM*, *PHB*, and *ERF022* of regulatory roles in plant regeneration to verify this assumption. Three barley genotypes that differed in capacity for plant regeneration were involved in gene expression profiling, including GP of efficient plant regeneration and poorly regenerating genotypes “Scarlett” and “Dema.”

**Fig. 3.**
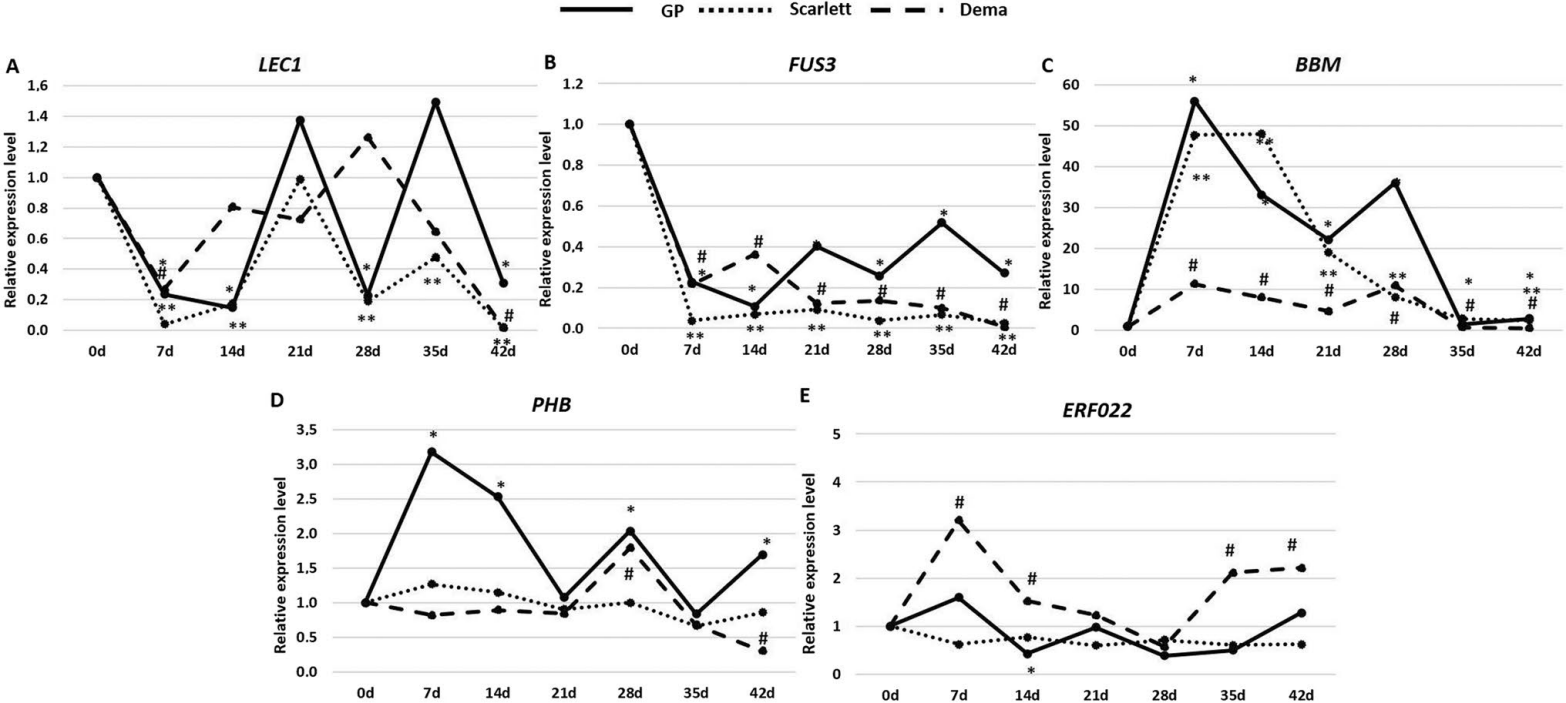
Expression pattern of plant regeneration-related TF genes, including *LEC1* (**A**), *FUS3* (**B**), *BBM* (**C**), *PHB* (**D**), and *ERF022* (**E**), in the barley cultures of different plant regeneration potential, including “Golden Promise” of high regeneration potential and two genotypes poorly responding in vitro, “Scarlett” and “Dema”. * — values significantly different from 0 day of GP (*p* < 0.05; *n* = 3; means ± SD are given); ** — values significantly different from 0 day of “Scarlett” (*p* < 0.05; *n* = 3; means ± SD are given); # — values significantly different from 0 day of “Dema” (*p* < 0.05; *n* = 3; means ± SD are given)

The expression level of genes was analyzed at different time points of explant culture and compared to that in 0 day explants (Fig. 3). All genotypes showed significantly modulated expression of the genes, including *LEC1*, *FUS3*, *BBM*, *PHB*, and *ERF022*, during the culture. The increase and decrease in the gene expression were indicated to be gene- and genotype-dependent. Some similarities in gene expression patterns in different genotypes might be noticed. For example, we noted that the explant induction on the CIM medium decreased the expression of *LEC1* and *FUS3* and increased *BBM* transcription in the early culture (7 days) in all analyzed genotypes.

The pattern of *ERF022* expression in the culture of “Dema” and “Scarlett” showed some similarity to that in GP in most of the analyzed culture time points (Fig. 3E). In contrast, the *PHB* gene revealed a specific pattern of expression in GP culture. The level of *PHB* transcript was similar to 0 day explants over the “Scarlett” and “Dema” cultures course, while it was significantly modulated in GP (Fig. 3D).

**Fig. 4.**
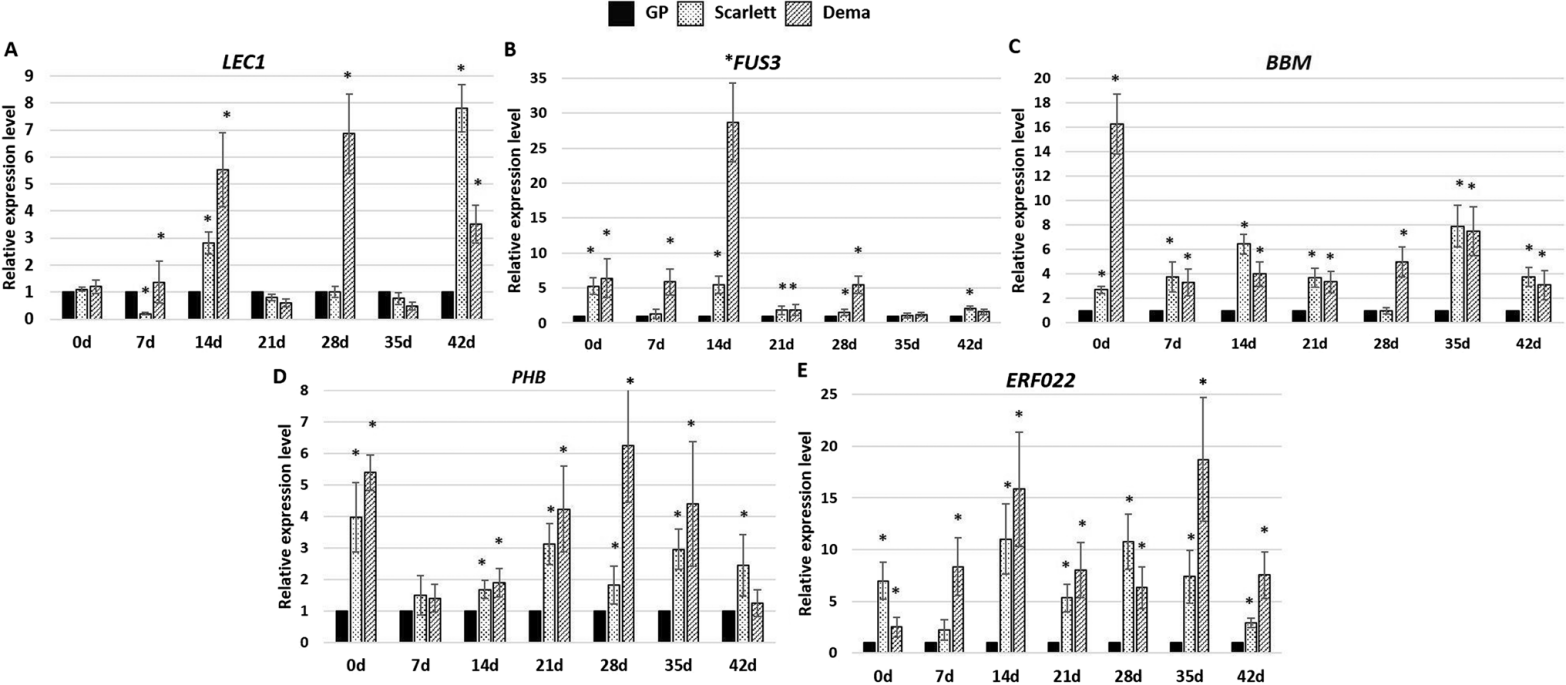
The expression level of TF genes *LEC1* (**A**), *FUS3* (**B**), *BBM* (**C**), *PHB* (**D**), and *ERF022* (**E**) in poorly responding in vitro genotypes of “Scarlett” and “Dema” in relevance to “Golden Promise” of high regeneration potential. The expression level of genes in “Scarlett” and “Dema” cultures was calibrated to expression in GP culture at the same age. * — values significantly different from “Golden Promise” at the same time point (*p* < 0.05; *n* = 3; means ± SD are given)

To get better insight into the relationship between plant regeneration capacity and *TF* gene expression, “Scarlett” and “Dema” transcript levels were compared to those indicated on the relevant day of GP culture with high plant regeneration capacity. The analysis showed a significantly higher expression level (from 1.5 to 30 times) of all genes (*FUS3*, *LEC1*, *BBM*, *ERF022*, *PHB*) in plant regeneration recalcitrant genotypes of “Dema” and “Scarlett” than in the GP culture (Fig. 4). Similar to the cultured explants, also the freshly isolated explants (0 day) of “Dema” and “Scarlett” showed a significantly higher (from two to 16 times) than GP accumulation of transcripts of all genes except for *LEC1*. Thus, over-optimal expression levels of genes controlling plant morphogenesis in vitro might be related to a low regeneration potential of in vitro recalcitrant barley genotypes, “Dema” and “Scarlett”.

Concluding, barley cultures of different genotypes showed differential expression of plant regeneration-related *TF* genes, including *LEC1*, *FUS3*, *BBM*, *PHB*, and *ERF022*. Noteworthy, a higher level of TF transcripts in freshly isolated and cultured explants was characteristic of poorly plant-regenerated genotypes (“Dema” and “Scarlett”) compared to GP.

#### Barley genotypes differ in the content of phytohormones in both explants and explant-derived cultures

**Fig. 5.**
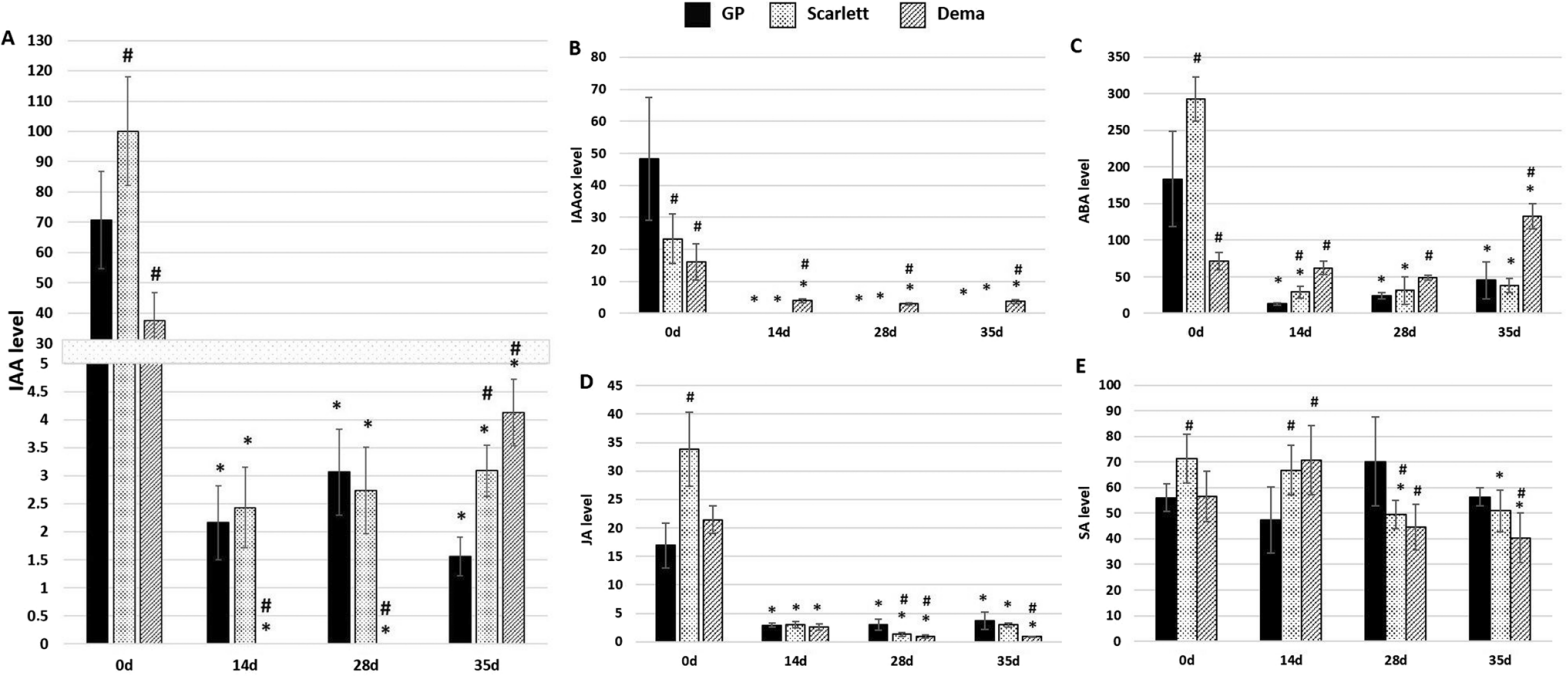
Accumulation of different hormones (ng/gDW), including free-IAA (**A**), IAAox (**B**), ABA (**C**), JA (**D**), and SA (**E**) in freshly isolated (0 day) in vitro and cultured barley explants on CIM medium (14 days, 28 days) and PRM medium (35 days) of different genotypes: “Golden Promise”, “Scarlett”, and “Dema”. DW, dry weight. * — values significantly different from 0 day explants of the same genotype (*p* < 0.05; *n* = 3; means ± SD are given); # — values significantly different from the “Golden Promise” at the same culture time point (*p* < 0.05; *n* = 3; means ± SD are given)

The content of auxin (free IAA) and its primary catabolite (IAAox), together with stress phytohormones (ABA, JA, and SA), were evaluated in barley cultures of different genotypes. Phytohormones were quantified in the freshly isolated (0 day) and in vitro cultured for 14, 28, and 35 days explants of GP, “Scarlett”, and “Dema” (Fig. 5A–E).

The results showed that a common response of all genotypes to in vitro culture conditions was a rapid and significant decrease of auxin (free IAA), its catabolite IAAox, and two of the stress hormones, JA and ABA, in the cultured explants (Fig. 5A–D). The results also indicated that the primary (0 day) and cultured explants of poorly regenerating genotypes of “Dema” and “Scarlett” significantly differed from GP explants in the content of free IAA. Accordingly, the freshly isolated explants of “Scarlett” accumulated up to 1.4 times more free IAA than the GP explants. In contrast, 0 day explants of “Dema” contained a distinctly lower (1.9 times) than GP level of free IAA. In cultured explants, the difference in accumulation of IAA was strongly pronounced in “Dema” compared to GP. The specific response of “Dema” explants was a lack of free IAA on the CIM medium and the accumulation of IAAox catabolite in explants induced on both CIM and PRM. In “Scarlett”, a level of free IAA on the CIM medium was similar to GP, while explant transfer onto PRM medium (35 days) resulted in elevated over GP accumulation of auxin.

Besides auxin, levels of stress hormones showed distinct differences between genotypes. Compared to GP, the primary explants (0 day) of “Scarlett” contained more ABA (1.6 times) and JA (two times), while in “Dema”, the level of ABA was lower (2.5 times). Genotype-dependent differences in stress hormone levels were also found in explants cultured on CIM and PRM media. For example, in “Dema” culture, the content of JA was lower (over three times), while ABA accumulation was higher (from 1.2 to 3.9 times) than in the GP culture.

We found that the content of auxin and stress-phytohormones (ABA, JA, and SA) both in primary and in vitro cultured explants significantly differed between barley genotypes suggesting that genotype-specific control of phytohormones accumulation might contribute to a strong effect of genotype on plant regeneration in barley. Regardless of genotype, a common response of explants to culture media was a substantial decline in IAA, ABA, and JA content.

#### TSA treatment improves the regeneration potential in some barley genotypes

**Fig. 6.**
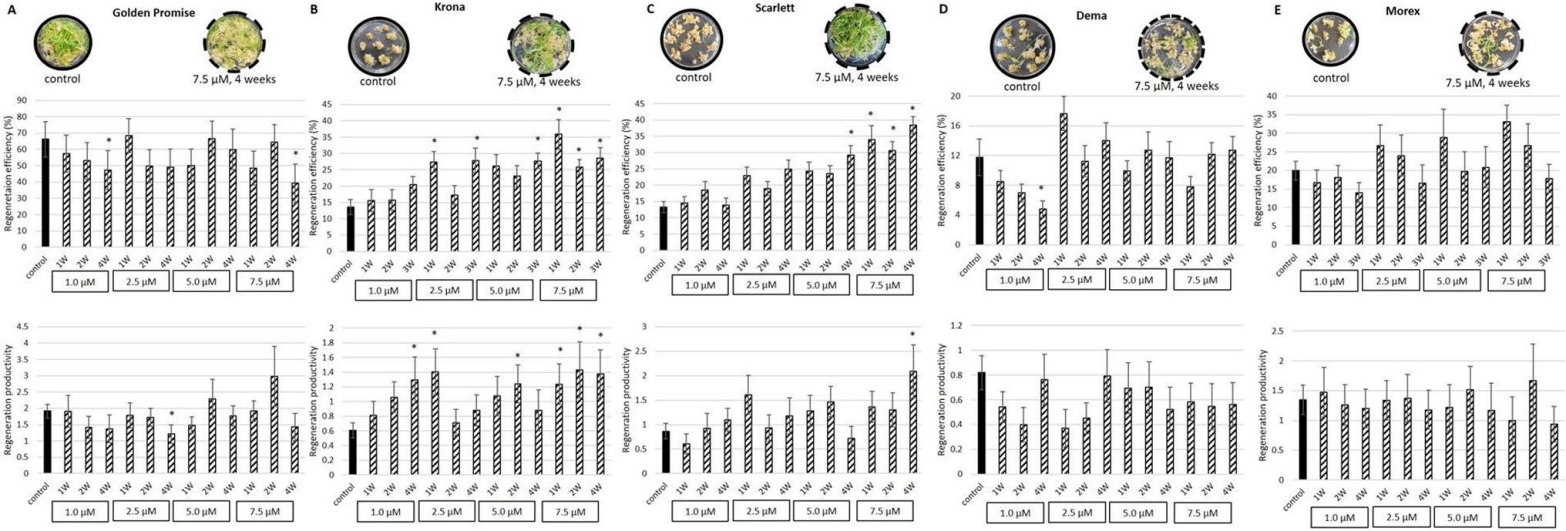
Effect of TSA treatment on plant regeneration in different barley cultivars: “Golden Promise,” “Krona,” “Scarlett,” “Dema,” and “Morex.” Plant regeneration efficiency and productivity evaluated in barley cultures treated with 0.0, 1.0, 2.5, 5.0, and 7.5 μM of TSA for different times (1, 2, and 4 weeks) in different barley genotypes: “Golden Promise” (**A**), “Krona” (**B**), “Scarlett” (**C**), “Dema” (**D**), “Morex” (**E**). W, weeks. * — values significantly different from the control (untreated with TSA) culture (*p* < 0.05; *n* = 3; means ± SD are given)

To evaluate the effect of TSA on plant regeneration in barley, the explants of five barley genotypes were cultured on a CIM supplemented with different (0.0, 1.0, 2.5, 5.0, and 7.5 μM) TSA concentrations. TSA treatment significantly increased plant regeneration effectiveness (both efficiency and productivity) in two out of five treated barley genotypes. Accordingly, the explants of “Krona” and “Scarlett” treated with 7.5 μM of TSA showed up to two times higher efficiency and productivity of plant regeneration (Fig. 6). Interestingly, explant treatment with 7.5 μM of TSA for 1, 2, and 4 weeks similarly affected plant regeneration. This result might suggest a central role of histone acetylation in regulating plant regeneration-related genes at the very early stage of culture when transcriptomic reprogramming of somatic explant cells is induced.

In contrast to the beneficial effect in “Krona” and “Scarlett”, TSA treatment negatively affected plant regeneration in GP and “Dema”. Accordingly, treatments with 1.0 and 2.5 μM TSA for 4 weeks substantially decreased the plant regeneration potential of these genotypes. In “Morex” culture, TSA treatment resulted in no significant changes in plant regeneration capacity.

The results showed a strongly genotype-dependent effect of TSA treatment on plant regeneration in barley. In two genotypes, “Scarlett” and “Krona” TSA treatment resulted in a higher number of regenerated plants suggesting that modification of the epigenetic status of explant cells with TSA might improve the plant regeneration efficiency in some recalcitrant barley genotypes.

#### TSA treatment increases the expression of plant regeneration-related genes in the barley culture

**Fig. 7.**
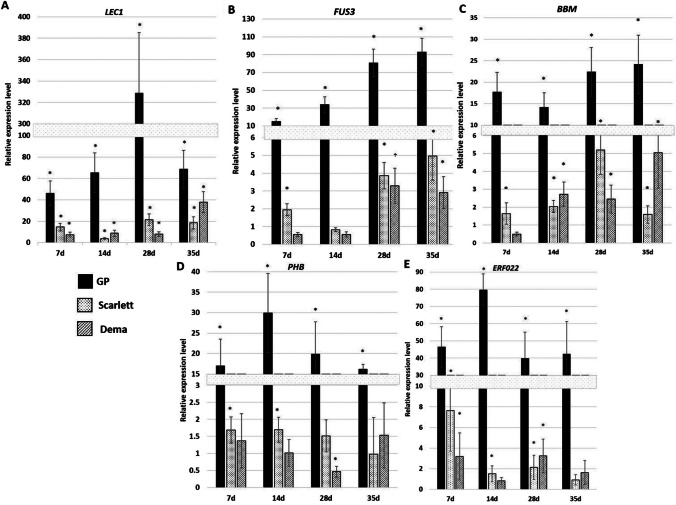
TSA-induced increased expression level of the plant regeneration-related TF genes: *LEC1* (**A**), *FUS3* (**B**), *BBM* (**C**), *PHB* (**D**), and *ERF022* (**E**) in the culture of barley cultivars: “Golden Promise”, “Scarlett”, and “Dema”. Explants were treated with 7.5 μM of TSA for 4 weeks. The expression level of genes in the TSA-treated culture was calibrated to expression in the untreated (control) culture of the same genotype. * — values significantly different from the control culture (untreated with TSA) of the same genotype at the same time point (*p* < 0.05; *n* = 3; means ± SD are given)

Given that TSA might affect plant regeneration via controlling gene expression, we analyzed the effect of TSA treatment on expression levels of the plant regeneration genes in the culture of GP, “Dema”, and “Scarlett.” The results indicated that TSA treatment stimulated expression of all analyzed *TF* genes (*LEC1*, *FUS3*, *BBM*, *PHB*, *ERF022*), and the level of TSA-induced increase of expression varied between genes, genotypes, and tissue culture stages (Fig. 7). The highest stimulation of gene transcription was indicated in GP culture in which most of the genes, including *FUS3*, *BBM*, *ERF022*, and *PHB*, enhanced the transcription up from 10 to 92 folds, while the maximal transcript stimulation, up to 330 folds, was indicated for *LEC1*. In “Scarlett” and “Dema” cultures, the stimulatory effect of TSA on the gene expression was less pronounced and ranged from 2 folds for *FUS3* to 21 folds for *LEC1*.

## Discussion

### Diverse capacity for plant regeneration of the barley genotypes

The current challenges in cereal and barley biotechnology involve the identification of the genotype-related determinants of plant regeneration in barley and the development of new experimental approaches that enable the efficient regeneration of plants from transformed tissue of different genotypes (Matres et al. 2021). To this end, we analyzed *in vitro *responses of different barley genotypes, including GP and four malting barley cultivars, “Dema”, “Scarlett”, “Morex”, and “Krona”. These cultivars originated from different parents (see M&M) and showed a high morphological and physiological diversity between them and in comparison to GP (https://barley.agricrops.org/cultivars/compare; Horvath et al. 2003; Cantalapiedra et al. 2017). Noteworthy, the “Morex” was used to create a mapping population (Rao et al. 2007), and the genome was sequenced (Beier et al. 2017).

The results showed a diverse capacity for plant regeneration among the analyzed barley genotypes. In contrast to GP, which regenerated 1.9 plants per explant with an efficiency of 65%, the malting cultivars indicated much lower efficiency (12–18%) and productivity (0.6–1.3) of plant regeneration. Similar to our results, low plant regeneration productivity in “Scarlett”, “Krona”, and “Morex” cultures was also reported by others where IZEs were induced on different media (Chang et al. 2003; Halámková et al. 2004; Hisano et al. 2016). The poor plant regeneration of malting cultivars corresponds with other studies reporting that the efficiency of plant regeneration in barley ranged from 3 to 33% depending on cultivar and regeneration protocol (Chang et al. 2003; Halámková et al. 2004; Aguado-Santacruz et al. 2011; Gubišová et al. 2012; Ślesak et al. 2013). Rarely, manipulation with media composition, mainly PGRs, might improve the plant regeneration of the culture (Przetakiewicz et al. 2003). Adjusting genotype-specific media composition promoting plant regeneration seems time-consuming, costly, and mostly unsuccessful. Thus, to overcome in vitro barley recalcitrance, identifying genetic and epigenetic factors controlling plant regeneration processes and developing new experimental approaches is of central interest to modern biotechnology of this important crop.

### Two alternative morphogenic pathways, SE and shoot ORG, contribute to in vitro regeneration of barley plants

Two alternative morphogenic processes, SE and shoot ORG, contribute to plant regeneration in vitro (Bidabadi and Mohan Jain 2020). Depending on the plant species, explant type, and culture protocol, the regenerants are usually produced in favor of one of these pathways (Alemanno et al. 1996; Li et al. 2013; Bartos et al. 2018; Jung et al. 2021). Detailed histological analysis of barley cultures has rarely been conducted, and mostly somatic embryo-like structure development has been reported in cultures derived from mature and immature barley zygotic embryos (Kachhwaha et al. 1997; Nonohay et al. 1999; Eudes et al. 2003).

The histological analysis of the GP culture revealed that both somatic embryos and shoot meristems were produced in the callus suggesting two alternative pathways, SE and ORG, were involved in the regeneration of barley plants. The contribution of ORG and SE has also been reported in barley cultures derived from mature and IZEs induced on different media (Bouamama et al. 2011; Ślesak et al. 2013). Similarly, in other plants, including monocots (Suzuki and Nakano 2001; Mazri et al. 2018) and dicots (Jach and Przywara 2000; Tomaszewska-Sowa et al. 2017; Liang et al. 2020), shoot induction might accompany somatic embryo development. Simultaneous induction of different morphogenic pathways might suggest at least partial similarity of the regulatory network controlling plant regeneration via ORG and SE.

### Genotype-dependent capacity for plant regeneration in barley is associated with differential expression of auxin-related *TF* genes

The overexpression of SE-related TFs such as *BBM*, *LEC1*, *LEC2*, *WUS*, and *WIND1* stimulated the plant regeneration in different plants, including those in vitro recalcitrant, suggesting a central role of genetic regulators in controlling plant regeneration (Srinivasan et al. 2007; Deng et al. 2009; Iwase et al. 2011; Heidmann et al. 2011; Ikeuchi et al. 2013; Horstmann et al. 2017). Most *TF* genes controlling plant regeneration were attributed to the metabolism and signaling of plant phytohormones (Salaün et al. 2021). Thus, to get insights into the genotype-dependent capacity for plant regeneration in barley, we analyzed the expression of hormone-related *TF* genes of an essential function in SE induction in Arabidopsis, including *LEC1*, *BBM*, *FUS3*, *ERF022*, and *PHB* (Gaj et al. 2005; Braybrook et al. 2006; Nowak et al. 2015; Wójcik et al. 2017). Besides SE, the role of *BBM*, *LEC1*, *FUS3*, and *PHB* in ORG suggested common functions of the studied TFs in plant regeneration processes (Horstmann et al. 2017; Tang et al. 2017; Zhang et al. 2017; Ikeda et al. 2020; Raspor et al. 2021).

Three barley genotypes of contrasting capacities for plant regeneration, a highly in vitro potent GP cultivar, and two poorly responding cultivars, “Scarlett” and “Dema”, were chosen for the *TF* expression analysis. The results indicated the high diversity of *TF* transcript levels between the genotypes in the explants (0 day) and the derived cultures. Interestingly, the TF expression levels were significantly higher in the “Dema” and “Scarlett” than in the GP culture of efficient plant regeneration. We also found that the expression patterns of analyzed *TFs* were modulated differently over culture time in barley cultures of different genotypes. However, some similarities in expression patterns of *TFs* were indicated, including decreased expression of *LEC1* and *FUS3* and increased *BBM* transcription in the early barley cultures of different genotypes. Since *BBM* positively regulates *LEC1* and *FUS3*, the members of *LAFL* (*LEC2,* *ABI3*, *FUS3*, *L1L*) genes (Horstman et al. 2017), increased expression of *LEC1* and *FUS3* was associated with the upregulation of *BBM* in cultures of different species (Florez et al. 2015; Grzybkowska et al. 2018; Brand et al. 2019; Awada et al. 2023). In contrast, we indicated that in barley cultures, the upregulation of *BBM* was accompanied by decreased expression of *LEC1* and *FUS3,* suggesting differences in regulatory interactions between these TFs in the genetic network controlling plant regeneration of barley and other species.

The genotype-specific expressions of the *LEC1* and *BBM* in barley cultures were also reported by others (Orłowska et al. 2017; Wen et al. 2020; Kumar et al. 2021; Suo et al. 2021). Moreover, global transcriptomic analyses of barley cultures derived from GP and in vitro recalcitrant genotypes indicated numerous differentially expressed genes (DEGs) that might be related to plant regeneration potential (Suo et al. 2021; Xu et al. 2022). Noteworthy, the DEGs involved transcripts that were up- and down-regulated in GP in relevance to the poorly responding barley genotype (Suo et al. 2021; Xu et al. 2022). Significantly, a top group of DEGs was enriched in genes involved in auxin biosynthesis (*YUCCA*), signaling (*ARFs* and *Aux/IAAs*), and polar auxin transport (*PIN*s), pointing to a central role of auxin in plant regeneration of barley (Suo et al. 2021; Xu et al. 2022). In support of this postulate, *BBM*, *LEC1*, and *FUS3* TFs that we also found differentially expressed in barley, control auxin-related genes, including *YUC*, *Aux/IAA*, *ARF*, and *PIN*, and regulate embryogenic induction in Arabidopsis (Yamamoto et al. 2009; Junker et al. 2012; Wang and Perry 2013; Horstmann et al. 2017). These results suggest that *BBM*, *LEC1*, and *FUS3* can be considered as candidate TFs for further research on auxin-related genetic pathways regulating plant regeneration potential in barley. In Arabidopsis, these TFs interact with LEC2 of the B3 TF gene family, which is essential in auxin-dependent embryogenic transition (Horstmann et al. 2017). Surprisingly, *LEC2* has not been identified in the barley genome (databases: EnsemblPlants, Plaza), and that implies some differences in the genetic regulation of auxin-related embryogenic induction in barley and Arabidopsis. These differences might involve the genetic interactions of *PHB* and *ERF022*, which in SE of Arabidopsis operate in the LEC2-related pathways (Nowak et al. 2015; Wójcik et al. 2017).

In support of the role of auxin in the genotypic diversity of plant regeneration capacity in barley, we indicated distinct differences in auxin accumulation (free IAA and IAAox) in explants and explant-derived callus cultures of the GP, “Dema”, and “Scarlett”. Similarly, callus cultures of other in vitro recalcitrant barley cultivars (“Haruna Nijo” and “Morex”) accumulated IAA at a level different from the GP culture (Hisano et al. 2016). Although the links between auxin content in explants/callus and the plant regeneration potential were also reported in other plant species (Nic-Can and Loyola-Vargas 2016), in vitro responding genotypes indicated both higher and lower accumulation of IAA than those in vitro recalcitrant (Żur et al. 2015; Hisano et al. 2016; Grzyb et al. 2017; Caeiro et al. 2022).

To control plant development, auxin interacts with other phytohormones, including those related to stress responses, which are essential in in vitro induced plant morphogenesis (Zavattieri et al. 2010; Fehér 2015). Relevantly, numerous stress-related genes, including those related to stress hormones, were differentially expressed in embryogenic cultures of different plants (Legrand et al. 2007; Wickramasuriya and Dunwell 2015), including barley cultures of opposed plant regeneration potential (Xu et al. 2022). Similarly, we indicated different accumulations of ABA, JA, and SA in GP vs. “Dema” and “Scarlett” cultures. The relationship between hormone content and plant regeneration capacity seems to be complex and inconclusive results on phytohormone ratio, including IAA/ABA, favoring plant regeneration were reported in barley (Hisano et al. 2016) and other plants (Centeno et al. 1997; Zhou et al. 2017; Gatica-Arias et al. 2019; Awada et al. 2020). Altogether, the results provide evidence that the genotype-specific control of phytohormones levels might contribute to the diverse capacities of barley cultivars for plant regeneration.

We profiled the expressions of auxin biosynthesis-related genes to get insights into metabolic pathways controlling auxin accumulation in barley cultures. The results indicated *HvYUC7/AtYUC10* (Harb et al. 2020) and *TAA1* (*TRYPTOPHAN AMINOTRANSFERASE OF ARABIDOPSIS 1*) genes to be differentially expressed in “Dema” and “Scarlett” in comparison to GP culture (Supplementary Fig. 1). Both genes have a critical role in auxin biosynthesis via the Trp (tryptophan)-dependent IPyA (indole-3-pyruvic acid) pathway in plants, including barley (Pérez-Pérez et al. 2019). The role of *TAA1* and different *YUC* genes, including *YUC10*, in IAA biosynthesis in plant morphogenesis in vitro was indicated (Bai et al. 2013; Wójcikowska et al. 2013; Raspor et al. 2021). Thus, we assumed that the IPyA pathway might also be involved in auxin biosynthesis associated with the regeneration of barley plants. Further analysis is required to identify the components of the Trp-dependent IPA pathway, including other genes of the eight members of the *YUC* gene family in barley (Harb et al. 2020) that might be involved in controlling auxin biosynthesis in plant regeneration in this crop.

### TSA modifies the expression of genes and improves the plant regeneration potential of poorly responding in vitro barley genotypes

Distinct differences in the expression of *TF* genes found between the GP and poorly in vitro responding genotypes suggested that modifying gene expression might improve plant regeneration in barley. Following this assumption, we treated the barley explants with TSA, the chromatin modifier which, via inhibition of TSA-sensitive HDACs, changes histone acetylation and gene expression (Görisch et al. 2005).

Our results showed the genotype-specific effect of TSA in barley cultures. The stimulatory effect of the TSA treatment on plant regeneration was indicated in two barley cultivars, “Scarlett” and “Krona”. The genotype-dependent results of TSA treatment were also observed in *B. rapa* (Zhang et al. 2016) and wheat (Jiang et al. 2017; Bie et al. 2020). Besides genotype, the effect of TSA on plant regeneration depends on treatment conditions such as concentration and treatment time (Castillo et al. 2020; Martínez et al. 2021; Choi et al. 2023). We tested the effect of TSA at a wide range of concentrations (1.0–7.5 μM) and durations of treatment (1, 2, and 4 weeks) and indicated the dose of 7.5 μM of TSA for 4 weeks to be effective in stimulation of plant regeneration in some barley genotypes. In other plants, TSA in the concentration of 0.1–2.5 μM for 10 min–3 weeks was used (Jiang et al. 2017; Wójcikowska et al. 2018; Castillo et al. 2020; Bie et al. 2020). In wheat, depending on genotype, microspores treatment with 0.1 and 0.3 μM of TSA for 10 min was effective in plant regeneration stimulation (Jiang et al. 2017). Altogether, the results indicated a high diversity of plant regeneration-effective TSA doses implying differences between species and genotypes in TSA sensitivity.

We assumed that TSA might affect plant regeneration in barley via stimulation of *TF* genes controlling plant regeneration. The results on gene expression profiling revealed that regardless of genotype, a common response to TSA treatment was the increase of *TF* gene transcripts level of *LEC1*, *FUS3*, *BBM*, *PHB*, and *ERF022* genes in barley cultures. Similarly, SE-involved *TFs* were upregulated in TSA-treated cultures of Arabidopsis (Wójcikowska et al. 2018), *P. abies* (Uddenberg et al. 2011), and *B. napus* (Li et al. 2014). Relevantly, the deregulation of a restricted set of genes was commonly reported due to TSA treatment (Lint et al. 1996; Chang and Pikaard 2005; Ma et al. 2016), implying a non-stochastic induction of genes by TSA (Xu et al. 2007). Thus, the analyzed TFs of regulatory function in plant regeneration seem to be specifically responsive to TSA. That implies the substantial role of histone acetylation and the TSA-sensitive HDACs in the genetic mechanism regulating plant regeneration. In support, the involvement of HDA6 and HDA19 in the repression of *LEC1*, *FUS3*, and *BBM* was reported (Tanaka et al. 2008; Jia et al. 2014; Morończyk et al. 2022). Thus, we assumed that the upregulation of *TFs* in barley cultures resulted from TSA-induced inhibition of the enzymatic activity of HDACs (Wójcikowska et al. 2018).

Besides inhibition of HDACs, TSA might affect cell transcriptome by other processes. For example, TSA might modulate the expression of *HDAC* genes, including those regulating plant-regeneration *TFs*. In support, we indicated intensive upregulation of *HDA6* and *HDA19* expression in response to TSA treatment in barley cultures of different genotypes (Supplementary Fig. 2). Similarly, TSA stimulated the expression of *HDAC*s, including *HDA6* in the culture of grapevine (Martínez et al. 2021), Arabidopsis (Morończyk et al. 2022), and wheat (Valero-Rubira et al. 2023). Relevantly, acetylation of histone marks has been indicated in the chromatin associated with the HAT/HDACs genes, including *HDA6* (PlantPan 3.0, Chow et al. 2019). Moreover, TSA-induced histone acetylation might impose other epigenetic changes, including histone methylation marks that activate (H3K27me3) and suppress (H3K4me2) gene expression (Pandey et al. 2016; Valero-Rubira et al. 2023). The reports on the diversity of the TSA-imposed epigenetic changes and the present results suggest the complex and genotype-specific effects of TSA on gene expression and plant regeneration.

The study demonstrated that treating barley explants with TSA affects the expression of *TFs* of regulatory function in plant regeneration and might provide an effective and simple method for improving plant regeneration response in some recalcitrant barley genotypes. Combining TSA treatment with other epigenetic agents, including DNA methylation inhibitor (5-Aza-deoxycytidine), might also be recommended to improve the developmental potential of in vitro recalcitrant species such as barley (Chang and Pikaard 2005; Wang et al. 2011). In conclusion, the wider implementation of epigenetic modifiers in research on in vitro plant regeneration might help to overcome problems hampering the common use of genetic transformation and genome editing in breeding important crop plants, including barley.

### Supplementary information

ESM 1

ESM 2

ESM 3

ESM 4
