# Supplementary figures and images for: The improvement of the in vitro plant regeneration in barley with the epigenetic modifier of histone acetylation, trichostatin A
Source: J Appl Genet. 2023 Nov 14;65(1):13–30. doi: 10.1007/s13353-023-00800-9 (PMC10789698; doi:10.1007/s13353-023-00800-9)

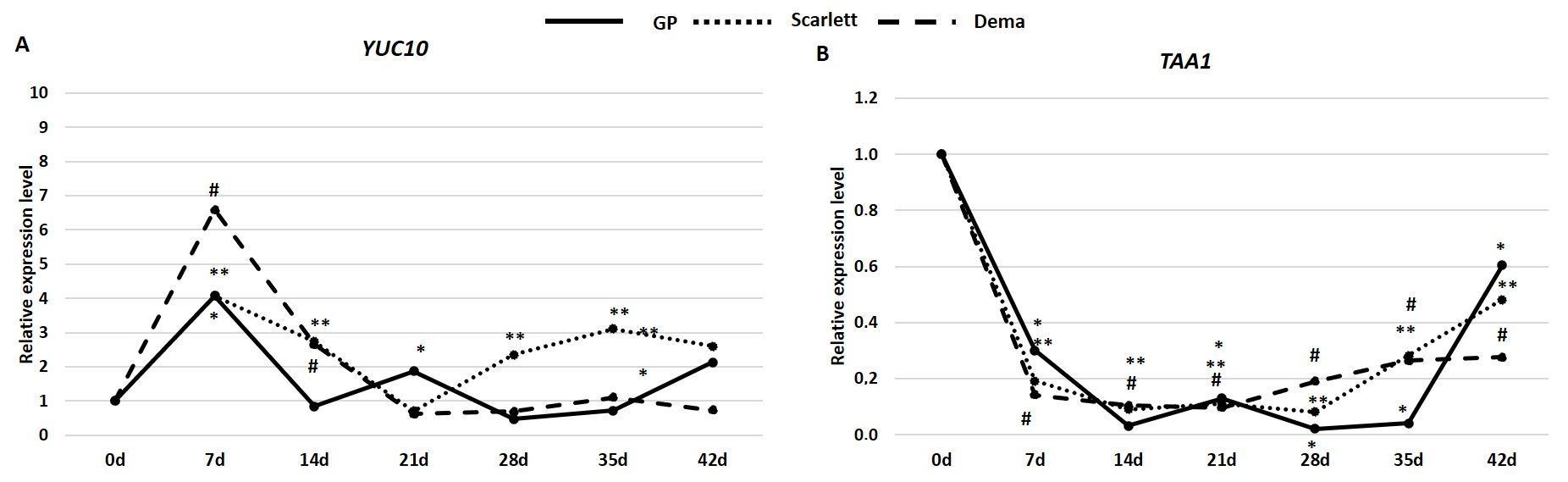

Supplement: Supplementary file 2 — ESM 2 [file 13353_2023_800_MOESM2_ESM.jpg]

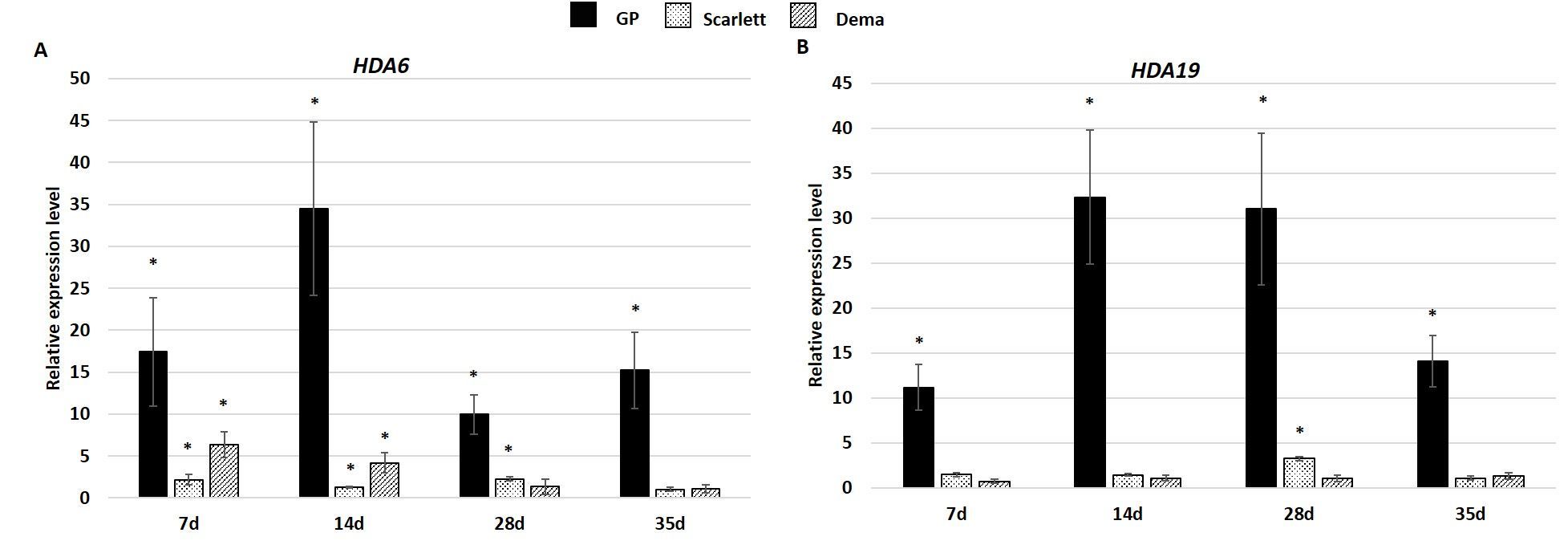

Supplement: Supplementary file 3 — ESM 3 [file 13353_2023_800_MOESM3_ESM.jpg]
